# Autogenous Transplantation for Replacing a Hopeless Tooth

**DOI:** 10.22037/iej.2017.22

**Published:** 2017

**Authors:** Mehrsa Zakershahrak, Amirabbas Moshari, Mehdi Vatanpour, Zohreh Khalilak, Afsoon Jalali Ara

**Affiliations:** a *General Dentist, Tehran, Iran; *; b * Department of Endodontics, Dental Branch, Islamic Azad University, Tehran, Iran*

**Keywords:** Autogenous, Auto-Transplantation, Endodontic, Surgical Procedure, Third Molar

## Abstract

Autogenous tooth transplantation (ATT) is a simple and reasonable choice for replacing the missing teeth when a proper donor tooth is available. This report presents a case of successful ATT of a maxillary right third molar for replacement of mandibular right second molar with a concomitant endodontic-periodontal disease. The mandibular second molar was believed to be hopeless due to a severe damage to coronal tooth structure, inappropriate root canal treatment and apical radiolucency. After extraction of mandibular second molar and maxillary third molar (the donor), the tooth was re-implanted into the extracted socket of second molar site. Root canal therapy was then performed. After 3 years, clinical and radiographic examinations revealed satisfying results, with no signs and symptoms. The patient is asymptomatic and the transplanted tooth is still functional with no signs of marginal periodontal pathosis. Radiographies showed bone regeneration in the site of previous extensive periapical lesion, normal periodontal ligament with no signs of root resorption.

## Introduction

Autogenous tooth transplantation (ATT) is the removal and transplantation of a tooth to an extracted-tooth socket or a surgically prepared alveolus in the same person [1, 2]. Abulcassis [3], documented the very first tooth transplantation in history in 1050 in ancient Egypt, where pharaohs forced their slaves to give their teeth to them. In 1564 Ambroise Par´e, a French dentist was the first person who recorded his surgery with details about tooth bud transplantation. Molar transplantation technique was described in 1956 and since then, the general guidelines for this surgical technique have remained almost the same [3, 4]. If a suitable donor tooth is available, ATT could be a substitute method to rebuild the arch. Due to the limited value of the donor tooth in the dentition, ATT of a third molar for replacing a lost first/second molar could be deliberated advantageous [1, 5].

Autogenous tooth transplantation is regularly done to replace the missing mature teeth, even with the prevalent use of dental implants [6-8]. It is quite a beneficial method, specifically for adolescents as implants and other prosthetic replacements may not be suitable [1, 9-11].

Improvement in aesthetics and mastication, maintenance of arch space integrity, preservation of bone volume and morphology, low cost and possibility of future orthodontic movement of the transplanted tooth are the benefits of this method [1, 5]. The result of ATT rests on careful case selection and deliberation of the biological rules as attainment of an apical seal is essential for normal function. In cases that the donor third molar has been transplanted before complete root formation, high success rates have been reported [12]. Furthermore, successful ATTs of teeth with complete root development have also been commonly reported [5]. ATT is achievable for molar teeth with closed apices and endodontic treatment is suggested. The donor tooth should be of limited value in the dentition, for example, a third molar.

This report demonstrates replacement of a mandibular second molar with a combined periodontal/endodontic lesion by performing a successful ATT with a maxillary third molar. 

**Figure 1:**
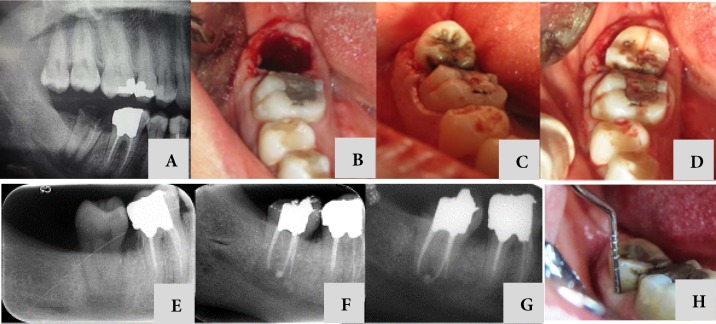
*A) *Radiography of mandibular left second molar (#47) and maxillary right third molar (#18); *B*) Socket of mandibular right second molar after irrigation with sterile saline; *C)* after transplantation (clinical view); *D) *After occlusal adjustment; *E) *Radiographic view after transplantation; *F) *Radiographic view after obturation; *G) *Radiographic view after 6 months; *H)* Normal periodontal probing after 6 months

## Case Report

A 28 year-old female patient with no contributing medical history and fair oral hygiene and chief complaint of seeking treatment for mandibular right second molar was referred to the Department of Endodontics of Islamic Azad University in 2012.

Extra-oral examination showed no facial swelling nor palpable lymph nodes. Intra-oral examination revealed no sinus tract and no periodontal pocket but the tooth’s clinical crown was severely destroyed. Prognosis of tooth #47 was hopeless due to severe damage of coronal tooth structure.

Radiographic examination showed unsatisfactory previous endodontic treatment with a large periapical lesion and broken lamina dura. Diagnosis of a combined periodontal and endodontic lesion was made. Clinical examination revealed a developed maxillary right third molar, which was completely erupted and was a proper candidate for ATT (Figure 1A). Complete descriptions of the procedure and its risks/benefits were presented to the patient and necessary permissions were obtained.

In the treatment session, 0.2% chlorhexidine gluconate solution was used for oral disinfection. Surgery was operated under local anesthesia with high tuberosity maxillary block for tooth #18 and inferior alveolar nerve block for tooth #47 using 2% lidocaine and 1:80000 epinephrine (Daroupakhsh, Tehran, Iran).

The mandibular right second molar was extracted atraumatically without any bone removal. Extraction of this tooth was done with a straight elevator and the socket was prepared by sterile saline irrigation. The maxillary right third molar was then extracted with forceps and was held less than 15 min in sterile wet gauze and then placed into the recipient socket. After transplanting the donor tooth, it was splinted with silk sutures and occlusal adjustments were made to remove the occlusal forces from the tooth (Figures 1B to E). 

The patient was ordered to use 0.12% chlorhexidine gluconate as mouth rinse, twice a day for 2 weeks. In addition, 400 mg ibuprofen TID (for 3days) was prescribed. 

The patient was revisited 5 days later to complete the treatment. In this session endodontic treatment was conducted. Root ZX electronic apex locator (J. Morita USA, Inc., Irvine, CA, USA) was used to determine the working length and was confirmed with x-ray.

Preparation of the root canals was performed with RaCe rotary instruments (FKG Dentaire, La-Chaux-de Fonds, Switzerland) using crown down technique. Final preparation of the canals was done with 35/0.04 in mesial canals and 40/0.04 in distal canal. After completion of cleaning and shaping of root canals a creamy mixture of calcium hydroxide was placed in to the root canals with lentulo spiral. The patient was revisited one month later. In the third visit, the treatment was completed with obturation of the mentioned tooth. Smear layer was removed with 5.25% NaOCl and 17% EDTA (Asia Chemi Teb. Co., Tehran, Iran). Lateral condensation technique was used for obturation of the canals and AH-26 (Dentsply, Tulsa Dental, Tulsa, OK, USA) was used as sealer (Figure 1F). Tooth crown was permanently restored with amalgam.

The patient was recalled for clinical and radiographic follow-ups after 1 day (post-operative check-up), 1 week (suture removal), 1 month (clinical examination) and 6 and 34 months (clinical/radiographic examinations). The patient was asymptomatic in the post-operative period (Figure 1G).

At the 3-year follow-up visit, the tooth had normal occlusion, physiological mobility and masticatory function; the tooth showed physiological/mesial migration to create normal contact with the adjacent first molar. Periodontal probing revealed no pockets or other pathological signs, there were no signs of attachment loss, and the patient was asymptomatic. Radiographic examinations showed a continuous intact periodontal ligament (PDL), bone regeneration in the site of previous periradicular radiolucency and no evidence of root resorption (Figure 1G and 1H).

## Discussion

Tooth transplantation can be categorized into several types: autogenous (where a tooth/tooth bud from one socket is placed into another socket in the same person), homogenous (if a person of the same specie as the receptor performs the donation) and heterogeneous (if the donor is from a different specie than the receptor) [1, 5]. Autogenous tooth transplantation or auto-transplantation is a tooth surgical transplantation from its original site in the mouth to another site in the same person [1].

In the 1950s, the success rate of autogenous tooth transplantation was about 50% because of the problem in estimating root resorption and its development after transplantation [13, 14]. This method (autogenous transplantation) was not common because of the lack of knowledge about the causes of root resorption and prevention methods. Since 1990, many studies have estimated the healing of periodontal diseases and the rate of dental root resorption after transplantation and the transplant success rate has increased [15-17]. Tsukiboshi [15] reported a success rate of 82% in 250 cases observed for 6 years. Lundberg and Isaksson [16] reported a success rate of 94% in cases with incomplete roots and 84% in cases with completely formed roots, while Mej`are *et al. *[17], reported high success rates for mature teeth. In this case, the transplanted molar had complete roots, which increased the transplantation success. Both clinical and radiographic results were successfully convincing in 6-month and 3-year follow ups.

Like other surgical procedures, for successful auto-transplantation, cautious case selection and treatment planning are important. The competency and proportion of the donor tooth and recipient site should both be precisely tested [18, 19]. The recipient site should have sufficient bone support with adequate attached keratinized tissue to allow tooth stabilization and also no inflammation or infection [18]. In the present case, the patient was presented with a mandibular right second molar tooth that was a candidate for extraction due to the presence of an extensive periradicular lesion and severely destroyed clinical crown. Presence of a healthy nearby maxillary right third molar with no opposing tooth provided an acceptable candidate for ATT. At three-year recall, there were no clinical signs and symptoms of infection and inflammation. In addition, no pain and no sensitivity to percussion, no root resorption and no ankylosis was observed. Follow-up radiographies after 6 months and three years, revealed bone healing at the site of previous periradicular radiolucency (at the recipient site), along with the presence of a continuous PDL space along the entire root surface. 

Inadequate buccolingual width in the recipient site or over preparation of this site may affect the alveolar ridge and result in resorption and loss of periodontal integrity. A general study on auto-transplantation of 53 molars with complete roots, reported that lack of buccal bone plate was the only significant analyst of the transplant failure [17]. In the present case, the recipient site had adequate bony support and keratinized mucosa to improve post-operative integrity.

Root canal therapy of teeth with closed apices is recommended 7 to 14 days after transplantation [20]. Usually, root canal therapy is completed a few weeks after transplantation [21]. Success rate may decrease due to the presence of a necrotic pulp and later infection that may cause inflammatory resorption; besides, infection at the host site may harmfully cause the failure of tooth transplantation. In the present case, orthograde root canal therapy was done 5 days after transplantation and then was completed 2 weeks later. In addition, there were no signs of infection at the site of maxillary right third molar and this tooth was vital and healthy.

The success of ATT may be ascribed to several factors including adequate root canal therapy and apical seal [22], preserving asepsis during the surgical procedure, atraumatic surgical extraction and replacement, minimum extra oral time, the shape of the recipient socket, PDL healing and vascularity of the recipient bed, proper occlusal interrelationship and sufficient bone support. In most cases, the preparation of the recipient socket requires more than 30 minutes [23]. Moreover, the procedure often includes fitting the tooth to the bone socket that may result in losing the periodontal ligament and root surface. Minimal manipulation of the donor tooth is necessary to save the Hertwig’s root sheath [1]; otherwise ankylosis or root resorption may happen. Successful transplantation involves good compatibility between the shape and size of donor tooth with the donor site. However, maxillary transplants have higher risk of failure due to the wide variation in size and shape of the teeth and the proximity of the maxillary sinus to the molar sockets [23]. 

In this case, extra oral time was minimum due to similar size of the donor tooth and the recipient site and their good compatibility. In addition, the donor tooth was protected carefully in a wet gauze for minimum handling and fitted in to the recipient site with minimum preparation, thus the procedure caused less trauma. Furthermore, the cervical compatibility between the tooth and bone reduced the chance of infection and increased the possibility of a suitable healing and periodontal reattachment. However, the transplanted tooth was fixed using sutures, because of the shape similarity between the tooth and bone socket, as most studies recommended flexible splinting for 7–10 days. This allowed functional movement and physiological mobility of the transplanted tooth and motivated and increased the periodontal ligament cells (*i.e*. fibroblasts, cementoblasts and osteoblasts) and bone repair [1, 23].

In the present case, after transplantation, occlusal reduction of the transplanted tooth was done to protect the tooth from any occlusal trauma and undesirable forces and to protect healing of the periradicular tissues.

Patient did not receive any prophylactic or postoperative antibiotics, as she did not suffer from any systemic diseases.

## Conclusion

ATT of third molars with complete root formation can be a clinically effective and economic treatment choice, compared to more complicated and expensive prosthetic/implant treatments. However, this is true only when with thorough case selection, atraumatic surgical methods, and good endodontic treatment can be performed by the operator. For further solid evidence, greater number of studies is required.
